# A retrospective study on the therapeutic effects of sodium bicarbonate for adult in-hospital cardiac arrest

**DOI:** 10.1038/s41598-021-91936-3

**Published:** 2021-06-11

**Authors:** Chih-Hung Wang, Cheng-Yi Wu, Meng-Che Wu, Wei-Tien Chang, Chien-Hua Huang, Min-Shan Tsai, Tsung-Chien Lu, Eric Chou, Yu-Lin Hsieh, Wen-Jone Chen

**Affiliations:** 1 Department of Emergency Medicine, Zhongzheng Dist, National Taiwan University Hospital, No.7, Zhongshan S. Rd, Taipei City, 100 Taiwan, ROC; 2 Department of Emergency Medicine, College of Medicine, National Taiwan University, Taipei City, Taiwan, ROC; 3 Department of Emergency Medicine, Baylor Scott and White All Saints Medical Center, Fort Worth, TX USA; 4 Department of Internal Medicine, Danbury Hospital, Danbury, CT USA; 5 Division of Cardiology, Department of Internal Medicine, National Taiwan University Hospital and National Taiwan University College of Medicine, Taipei City, Taiwan, ROC

**Keywords:** Cardiology, Health care

## Abstract

To investigate whether the effects of sodium bicarbonate (SB) during cardiopulmonary resuscitation (CPR) would be influenced by blood pH and administration timing. Adult patients experiencing in-hospital cardiac arrest (IHCA) from 2006 to 2015 were retrospectively screened. Early intra-arrest blood gas data were obtained within 10 min of CPR. Multivariable logistic regression analysis and generalised additive models were used for effect estimation and data exploration, respectively. A total of 1060 patients were included. Only 59 patients demonstrated favourable neurological status at hospital discharge. Blood pH ≤ 7.18 was inversely associated with favourable neurological outcome (odds ratio [OR], 0.24; 95% confidence interval [CI], 0.11–0.52; *p *value < 0.001) while SB use was not. In the interaction analysis for favourable neurological outcome, significant interactions were noted between SB use and time to SB (SB use × time to SB ≥ 20 min; OR 6.16; 95% CI 1.42–26.75; *p* value = 0.02). In the interaction analysis for survival to hospital discharge, significant interactions were noted between SB use and blood pH (Non-SB use × blood pH > 7.18; OR 1.56; 95% CI 1.01–2.41; *p* value = 0.05). SB should not be empirically administered for patients with IHCA since its effects may be influenced by blood pH and administration timing.

## Introduction

Annually, a total of 209,000 patients sustain in-hospital cardiac arrest (IHCA) in the United States^1^. Approximately 24% of patients with IHCA survive to hospital discharge; among these patients, 14% demonstrate severe neurological disability^1^.

Cardiac arrest may lead to severe acidosis^2–4^ because of increased anaerobic metabolism and cessation of pulmonary gas exchange. Buffering acidosis with sodium bicarbonate (SB) was intuitive; therefore, it had been recommended as a first‐line treatment for cardiopulmonary resuscitation (CPR)^5^. Nevertheless, SB administration during CPR was not without risks, such as worsening intracellular acidosis^6^, which may cause worse outcomes.

The guidelines^6,7^ did not recommend routine use of SB during CPR, except for hyperkalaemia-related arrest and tricyclic overdose. Using the American Heart Association Get With The Guidelines–Resuscitation database, Moskowitz et al.^8^ found a significant increase in SB use for IHCA between the years 2001 and 2016. Compared to the year 2001, patients with IHCA in the year 2016 had two-fold higher odds of receiving SB^8^, which resulted in half of them receiving SB during CPR^8^. This suggests SB may be used beyond the guideline-recommended indications^6,7^.

A small retrospective study^9^ revealed that approximately 31% (27 out of 88) of patients with IHCA received SB during CPR without any blood gas data and 11% (10 out of 88) of patients receiving the first SB injection empirically were later found to have alkalemia. Furthermore, a large retrospective study^10^ demonstrated that SB was often given late during CPR, probably as a “last-ditch effort,” which could have biased the results toward harmful association^11^. Therefore, it was probably not SB that worsened CPR outcomes. It just failed to improve outcomes.

Early blood gas analysis may reduce the frequency of unwarranted use of SB and clarify its effects during CPR. Therefore, in this exploratory analysis, we attempted to (1) investigate the therapeutic effects of SB during CPR, and (2) explored whether the effects of SB would be influenced by intra-arrest blood pH and timing of administration in the interaction analysis, thereby identifying the optimal indications for SB use during CPR.

## Materials and methods

### Setting

We used previously established IHCA database for analysis^12,13^. Briefly, this retrospective study was conducted at National Taiwan University Hospital (NTUH), a tertiary medical centre, which had 2600 beds, including 220 beds in intensive care units (ICUs). This study was performed in accordance with the Declaration of Helsinki amendments and approved by the Research Ethics Committee of the NTUH (reference number: 201805098RINC). The requirement for informed consent was waived because of the retrospective and non-interventional study design. In NTUH, a code team is activated whenever a cardiac arrest event occurs in the general wards. For IHCA in the ICUs, resuscitation is performed by the ICU staff without activating the code team. CPR is performed according to guideline recommendations^14,15^. In NTUH, point-of-care blood gas analysers are deployed in every ward floor, including floor of general wards and ICUs. Physicians are instructed to obtain blood samples as soon as possible for blood gas analysis in order to identify potential causes leading to IHCA.

### Participants

Patients sustaining IHCA at NTUH between 2006 and 2015 were screened by the following inclusion criteria: (1) age ≥ 18 years; (2) receiving CPR for ≥ 2 min; (3) absence of a do-not-resuscitate order before arrest and (4) early intra-arrest blood gas analysis with available blood pH, partial pressure of carbon dioxide (PCO_2_) and bicarbonate (HCO_3_^−^) data. If a single patient suffered from multiple IHCA events during hospitalisation, only the first event was analysed. Trauma-related IHCA was excluded. Patients were also excluded if the timing of SB use was not documented.

### Data collection and outcome measures

The following information was abstracted for each patient: age, sex, comorbidities, variables suggested by the Utstein template^16^, timing of SB use, early intra-arrest blood gas analysis data and critical interventions performed at the time of cardiac arrest and after sustained return of spontaneous circulation (ROSC). Sustained ROSC was defined as ROSC lasting for 20 min consecutively without resuming repeated CPR. Early intra-arrest blood gas analysis, including measurement of blood pH, PCO_2_ and HCO_3_^−^, was defined as the first available blood gas data measured within 10 min of initiating CPR, which was usually obtained in the beginning of CPR from the arterial source. Severe metabolic acidaemia was defined as blood pH ≤ 7.2, PaCO_2_ ≤ 45 mm Hg and HCO_3_^−^ concentration ≤ 20 mmol/ L^17^. Time to SB was defined as elapsed time from the first chest compression to the first time of SB administration. Duration of CPR was defined as the period from the initiation of chest compression to the termination of resuscitation attempts, either due to sustained ROSC or due to declaration of death.

The primary outcome was favourable neurological recovery at hospital discharge, defined as a Cerebral Performance Category scale score of 1 or 2^18^. The secondary outcome was survival to hospital discharge. The Cerebral Performance Category scale score was determined by retrospectively reviewing the medical records of each patient.

### Statistical analysis

Categorical variables are expressed as counts with percentages, while continuous variables are expressed as medians with interquartile ranges. Categorical and continuous variables were compared by Chi-squared test and Wilcoxon rank-sum test, respectively.

The odds ratio (OR) was designated as the outcome measure. Univariate and multivariable logistic regression analyses were conducted to investigate the associations between independent variables and outcomes. All available independent variables were accounted for in the regression model, regardless of whether they were considered as significant in univariate analyses. Generalised additive models (GAMs)^19^ were employed to explore nonlinear effects of the continuous variables on outcomes and to define the potential cut-off points for transforming a continuous variable into a categorical variable during the model-fitting process. The stepwise variable selection procedure with iterations between the forward and backward steps was utilized to build the final regression model. Significance levels for entry and to stay were defined at 0.15 to avoid exclusion of candidate variables. The final regression model was obtained by sequentially excluding individual variables with a *p *value > 0.05 until all regression coefficients were significant. If the variable of interest, SB use, was not included in the final model, this variable would be forcibly entered into the model to assess its effect estimate.

In the prespecified interaction analyses, the interactions of SB use with blood pH, severe metabolic acidaemia and time to SB were assessed. The goodness-of-fit of the fitted regression model was examined using *c* statistics, the adjusted generalised *R*^2^ and the Hosmer–Lemeshow goodness-of-fit test. Analysis was performed using R 3.3.1 software (R Foundation for Statistical Computing, Vienna, Austria). A two-tailed *p* value < 0.05 was considered significant.

## Results

Between 2006 and 2015, a total of 1698 adult non-trauma patients receiving CPR for ≥ 2 min after IHCA were screened. Of these, 599 patients were excluded due to the lack of early intra-arrest blood gas analysis; 39 patients were further excluded because the timing of SB use was not documented. Finally, a total of 1060 patients were included for further analysis. The differences between patients included and excluded from the analysis were shown in Supplemental Tables 1 and 2.

**Table 1:** Baseline Characteristics of Study Patients.

Variables	All Patients (n = 1060)	Patients With Favourable Neurological Outcome at Hospital Discharge (n = 59)	Patients Without Favourable Neurological Outcome at Hospital Discharge (n = 1001)	*p *value	Odds ratio (95% confidence interval)
Age, years, median (IQR)	68.2 (56.5–78.9)	62.6 (53.2–73.6)	68.5 (56.9–79.4)	0.007	0.98 (0.97–1.00)
Male, n (%)	649 (61.2)	44 (80.0)	605 (60.4)	0.03	1.92 (1.03–3.76)
Comorbidities, n (%)					
Heart failure, this admission	208 (19.6)	17 (28.8)	191 (19.1)	0.07	1.72 (0.90–3.16)
Heart failure, prior admission	171 (16.1)	11 (18.6)	160 (16.0)	0.59	1.20 (0.55–2.42)
Myocardial infarction, this admission	120 (11.3)	12 (20.3)	108 (10.8)	0.02	2.11 (0.99–4.20)
Myocardial infarction, prior admission	39 (3.7)	4 (6.8)	35 (3.5)	0.19	2.01 (0.50–5.91)
Arrhythmia	192 (18.1)	9 (15.3)	183 (18.3)	0.56	0.80 (0.34–1.69)
Hypotension	260 (24.5)	12 (20.3)	248 (24.8)	0.44	0.78 (0.37–1.51)
Respiratory insufficiency	764 (72.1)	37 (62.7)	727 (72.6)	0.10	0.63 (0.36–1.15)
Renal insufficiency	446 (42.1)	24 (40.7)	422 (42.2)	0.82	0.94 (0.53–1.65)
Hepatic insufficiency	182 (17.2)	8 (13.6)	174 (17.4)	0.45	0.75 (0.30–1.62)
Metabolic or electrolyte abnormality	186 (17.5)	8 (13.6)	178 (17.8)	0.41	0.73 (0.29–1.58)
Diabetes mellitus	354 (33.4)	17 (28.8)	337 (33.7)	0.44	0.80 (0.42–1.46)
Baseline evidence of motor, cognitive, or functional deficits	329 (31.0)	13 (22.0)	316 (31.6)	0.12	0.61 (0.30–1.17)
Acute stroke	45 (4.2)	1 (1.7)	44 (4.4)	0.32	0.38 (0.01–2.29)
Favourable neurological status 24 h before cardiac arrest	462 (43.6)	36 (61.0)	426 (42.6)	0.006	2.11 (1.20–3.79)
Pneumonia	337 (31.8)	8 (13.6)	329 (32.9)	0.002	0.32 (0.13–0.69)
Bacteraemia	86 (8.1)	0 (0)	86 (8.6)	0.02	0.09 (0.006–1.45)
Cirrhosis	70 (6.6)	1 (1.7)	69 (6.9)	0.12	0.23 (0.006–1.40)
Chronic obstructive pulmonary disease	62 (5.8)	4 (6.8)	58 (5.8)	0.75	1.18 (0.30–3.37)
Dialysis	191 (18.0)	10 (16.9)	81 (8.1)	0.83	0.92 (0.41–1.89)
Metastatic cancer or any blood-borne malignancy	247 (23.3)	4 (6.8)	243 (24.3)	0.002	0.23 (0.06–0.62)
Charlson comorbidity index, median (IQR)	2 (1–4)	2 (1–3.8)	2 (1–4)	0.004	0.83 (0.72–0.95)

Abbreviations: IQR, interquartile range.

**Table 2:** Features, Interventions and Outcomes of Cardiac Arrest Events.

Variables	All Patients (n = 1060)	Patients With Favourable Neurological Outcome at Hospital Discharge (n = 59)	Patients Without Favourable Neurological Outcome at Hospital Discharge (n = 1001)	*p *value	Odds ratio (95% confidence interval)
Arrest at night, n (%)	385 (36.3)	22 (37.3)	363 (36.3)	0.87	1.04 (0.58–1.85)
Arrest on weekend, n (%)	301 (28.4)	11 (18.6)	290 (29.0)	0.09	0.56 (0.26–1.12)
Arrest location, n (%)				0.06	1.05 (0.67–1.62)
Intensive care unit	473 (44.6)	29 (49.2)	444 (44.4)		
General ward	525 (49.5)	23 (39.0)	502 (50.1)		
Others	62 (5.8)	7 (11.9)	55 (5.5)		
Witnessed arrest, n (%)	734 (69.2)	43 (72.9)	691 (60.0)	0.53	1.21 (0.65–2.33)
Monitored status, n (%)	645 (60.8)	42 (71.2)	603 (60.2)	0.09	1.63 (0.89–3.10)
Shockable rhythm, n (%)	147 (13.9)	20 (33.9)	127 (12.7)	< 0.001	3.52 (1.88–6.42)
Critical care interventions in place at time of arrest, n (%)					
Mechanical ventilation	267 (25.2)	14 (23.7)	253 (25.3)	0.79	0.92 (0.46–1.74)
Antiarrhythmics	119 (11.2)	5 (8.5)	114 (11.4)	0.49	0.72 (0.22–1.84)
Vasopressors	474 (44.7)	21 (35.6)	453 (45.3)	0.15	0.67 (0.37–1.19)
Dialysis	78 (7.4)	3 (5.1)	75 (7.5)	0.49	0.66 (0.13–2.11)
Pulmonary artery catheter	6 (0.6)	2 (3.4)	4 (0.4)	0.003	8.70 (0.77–62.20)
Intra-aortic balloon pumping	8 (0.8)	1 (1.7)	7 (0.7)	0.39	2.45 (0.05–19.55)
CPR duration, min, median (IQR)	30 (14–49)	9 (5.3–18.8)	31 (15–50.3)	< 0.001	0.92 (0.90–0.95)
SB use, n (%)	733 (69.2)	27 (48.5)	706 (70.5)	< 0.001	0.35 (0.20–0.62)
Time to SB, median (IQR)	3.7 (0–6) (n = 733)	3.4 (0–4.5) (n = 27)	3.8 (0–6) (n = 706)	0.45	1.00 (0.96–1.05)
Intra-arrest blood gas analysis					
Blood pH, median (IQR)	7.2 (7.1–7.3)	7.3 (7.2–7.4)	7.2 (7.1–7.3)	< 0.001	14.30 (3.67–55.65)
PCO_2_, mmHg, median (IQR)	50.1 (35.0–74.0)	41.1 (32.3–57.0)	51.3 (35.0–75.0)	0.004	0.99 (0.97–1.00)
HCO_3_^−^, mmol/L, median (IQR)	20.2 (14.6–25.5)	20.7 (16.9–24.7)	20.1 (14.6–25.5)	0.43	1.00 (0.98–1.02)
Severe metabolic acidaemia, n (%)	115 (10.8)	5 (8.5)	110 (11.0)	0.55	0.75 (0.29–1.92)
Post-ROSC^e^ interventions, n (%)					
Extracorporeal membrane oxygenation	84 (7.9)	7 (11.9)	77 (7.7)	0.25	1.61 (0.60–3.73)
Targeted temperature management	12 (1.1)	3 (5.1)	9 (0.9)	0.003	5.88 (1.00–24.44)
Percutaneous coronary intervention	35 (3.3)	12 (20.3)	23 (2.3)	< 0.001	10.80 (4.60–24.29)
Sustained ROSC, n (%)	584 (55.1)	59 (100)	525 (52.4)	< 0.001	107.90 (6.65–1750.19)
Survival to hospital discharge, n (%)	124 (11.7)	59 (100)	65 (6.5)	< 0.001	1701.43 (104.01- 27,832.32)

Abbreviations: CPR, cardiopulmonary resuscitation; IQR, interquartile range; SB, sodium bicarbonate; PCO_2_, partial pressure of carbon dioxide; ROSC, return of spontaneous circulation.

The characteristics of the included patients are provided in Tables 1 and 2. Median patient age was 68.2 years. Median CPR duration was 30 min. A total of 733 patients (69.2%) received SB during CPR and the median time to SB was 3.7 min (The differences between patients with and without SB use were shown in Supplemental Tables 3 and 4). Median pH, PCO_2_ and HCO_3_^-^ levels were 7.2, 50.1 mm Hg and 20.2 mmol/L, respectively. There were 115 patients (10.8%) categorised as having severe metabolic acidaemia. Only 124 patients (11.7%) survived to hospital discharge; of these, 59 patients (5.6%) demonstrated favourable neurological status.

**Table 3:** Multiple Logistic Regression Model With Favourable Neurological Outcome at Hospital Discharge as the Dependent Variable.

Independent Variable^a^	Odds Ratio	95% Confidence Interval	*p* value
*Primary Model*			
CPR^b^ duration (min)	0.91	0.88–0.94	< 0.001
Post-ROSC^c^ percutaneous coronary intervention	10.47	3.97–27.60	< 0.001
Blood pH≦7.18	0.24	0.11–0.52	< 0.001
Favourable neurological status 24 h before cardiac arrest	2.80	1.46–5.38	0.002
Age (years)	0.97	0.95–0.99	0.002
Metastatic cancer or any blood-borne malignancy	0.18	0.06–0.55	0.003
Baseline evidence of motor, cognitive, or functional deficits	0.32	0.14–0.73	0.007
Post-ROSC targeted temperature management	9.46	1.66–54.01	0.01
Pneumonia	0.37	0.16–0.87	0.02
Pulmonary artery catheter in place at time of arrest	21.16	1.68–267.36	0.02
SB^d^ use	1.21	0.61–2.40	0.58
*Primary Model With Interaction Terms*			
CPR duration (min)	0.90	0.87–0.93	< 0.001
Post-ROSC percutaneous coronary intervention	10.81	4.07–28.69	< 0.001
Age between 23 and 69 (years)	3.40	1.73–6.70	< 0.001
Metastatic cancer or any blood-borne malignancy	0.15	0.05–0.48	0.001
Favourable neurological status 24 h before cardiac arrest	2.87	1.49–5.56	0.002
Non-SB use × blood pH ≦ 7.18	0.20	0.06–0.60	0.004
Baseline evidence of motor, cognitive, or functional deficits	0.30	0.13–0.70	0.005
SB use × blood pH ≦ 7.18	0.29	0.11–0.76	0.008
Post-ROSC targeted temperature management	11.74	1.68–81.93	0.01
SB use × time to SB≧20 (min)	6.16	1.42–26.75	0.02
Pneumonia	0.35	0.15–0.82	0.02
Pulmonary artery catheter in place at time of arrest	23.95	1.62–353.85	0.02

Primary model: goodness-of-fit assessment: n = 1060, adjusted generalised *R*^2^ = 0.40,estimated area under the receiver operating characteristic curve = 0.91, and Hosmer and Lemeshow goodness-of-fit Chi-Squared test *p* < 0.001; Primary model with interaction terms: goodness-of-fit assessment: n = 1060, adjusted generalised *R*^2^ = 0.43, estimated area under the receiver operating characteristic curve = 0.92, and Hosmer and Lemeshow goodness-of-fit Chi-Squared test *p* < 0.001.

^a^The display of independent variables is arranged in order of *p* value.

^b^CPR, cardiopulmonary resuscitation.

^c^ROSC, return of spontaneous circulation.

^d^SB, sodium bicarbonate.

**Table 4:** Multiple Logistic Regression Model With Survival at Hospital Discharge as the Dependent Variable.

Independent Variable^a^	Odds Ratio	95% Confidence Interval	*p* value
*Secondary Model*^b^			
CPR duration (min)	0.94	0.92–0.95	< 0.001
Post-ROSC percutaneous coronary intervention	5.80	2.44–13.80	< 0.001
Hypotension	0.36	0.20–0.64	< 0.001
Pulmonary artery catheter in place at time of arrest	24.56	3.12–193.32	0.002
Hepatic insufficiency	0.38	0.18–0.80	0.01
Shockable rhythm	1.94	1.13–3.34	0.02
Metastatic cancer or any blood-borne malignancy	0.48	0.26–0.89	0.02
Blood pH≦7.18	0.61	0.38–0.98	0.04
SB use	0.70	0.44–1.12	0.14
*Secondary Model With Interaction Terms*^c^			
CPR duration (min)	0.94	0.92–0.95	< 0.001
Post-ROSC percutaneous coronary intervention	5.16	2.15–12.39	< 0.001
Hypotension	0.37	0.21–0.67	0.001
Metastatic cancer or any blood-borne malignancy	0.44	0.24–0.81	0.003
Shockable rhythm	1.98	1.15–3.40	0.005
Pulmonary artery catheter in place at time of arrest	26.50	3.34–210.17	0.009
Hepatic insufficiency	0.40	0.19–0.83	0.009
Non-SB use × blood pH > 7.18	1.82	1.12–2.95	0.02
Favourable neurological status 24 h before cardiac arrest	1.56	1.01–2.41	0.05

Abbreviations: CPR, cardiopulmonary resuscitation; ROSC, return of spontaneous circulation; SB, sodium bicarbonate.

^a^The display of independent variables is arranged in order of *p* value.

^b^Secondary model: goodness-of-fit assessment: n = 1060, adjusted generalised *R*^2^ = 0.34,estimated area under the receiver operating characteristic curve = 0.85, and Hosmer and Lemeshow goodness-of-fit Chi-Squared test *p* = 0.10.

^c^Secondary model with interaction terms: goodness-of-fit assessment: n = 1060, adjusted generalised *R*^2^ = 0.36, estimated area under the receiver operating characteristic curve = 0.85, and Hosmer and Lemeshow goodness-of-fit Chi-Squared test *p* = 0.18.

**Figure 1:**
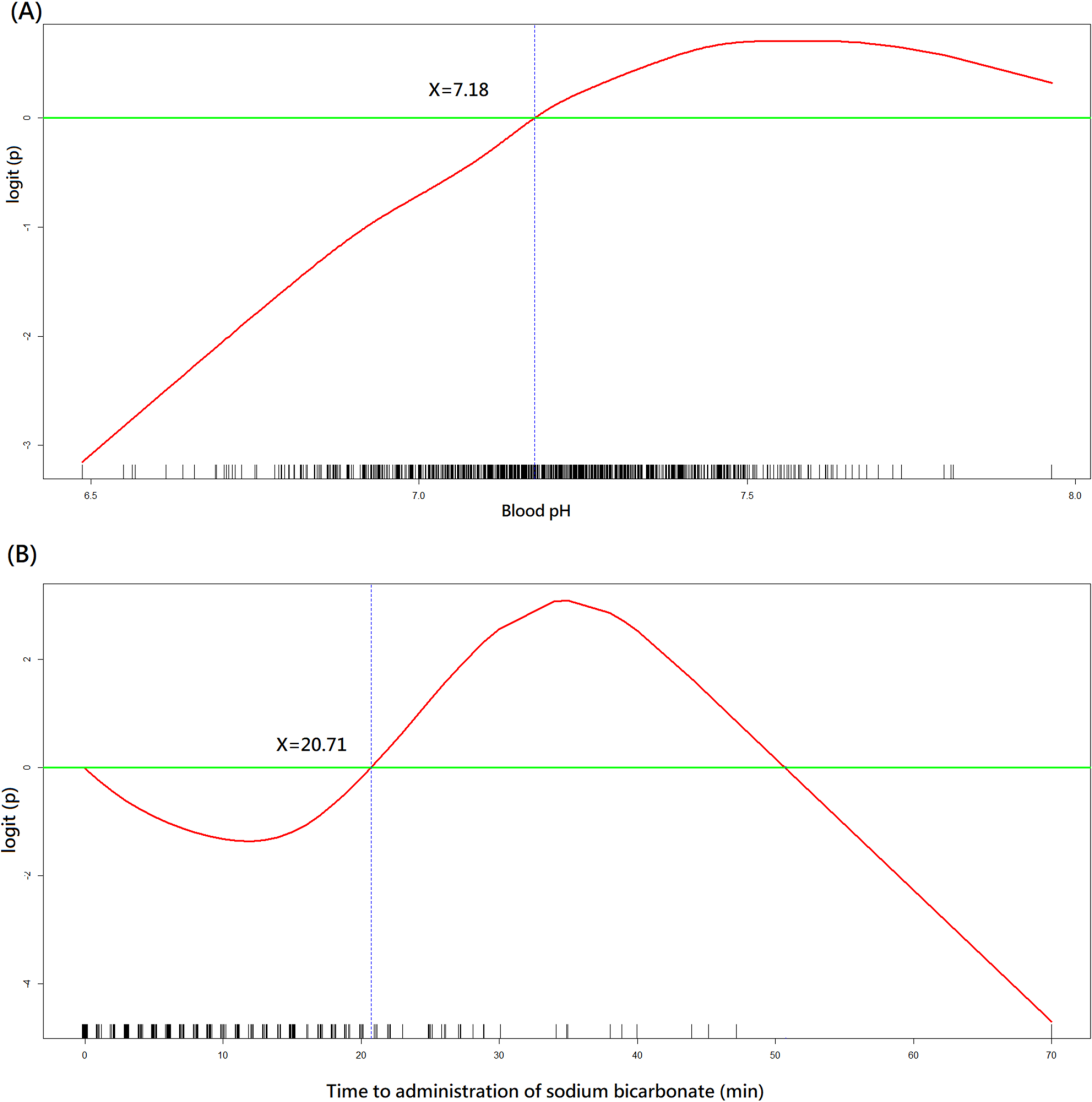
GAM plots. Nonparametric modelling of the effect of blood pH and time to administration of sodium bicarbonate on the logit of probability for favourable neurological outcome at hospital discharge. GAM, generalised additive model.

The GAM plots demonstrated the association of logit (p), where p represented the probability for favourable neurological status, with blood pH (Fig. 1A) and time to SB administration (Fig. 1B). If logit (p) was ˃0, the odds for favourable neurological status were ˃1. The cut-off points of blood pH value of 7.18 and time to SB of 20 min were used to transform blood pH and time to SB into binary variables during the model-fitting process.

For the primary outcome (Table 3), in the primary model, blood pH ≦ 7.18 was inversely associated with favourable neurological outcome (OR 0.24; 95% confidence interval [CI] 0.11–0.52; *p* value < 0.001) while SB use was not. In the interaction analysis, significant interactions were noted between SB use and blood pH (SB use × blood pH ≤ 7.18; OR 0.29; 95% CI 0.11–0.76; *p* value = 0.008; non-SB use × blood pH ≤ 7.18; OR, 0.20; 95% CI 0.06–0.60; *p *value = 0.004) or time to SB (SB use × time to SB ≥ 20 min; OR 6.16; 95% CI 1.42–26.75; *p *value = 0.02), but no significant interaction was noted between SB use and severe metabolic acidaemia.

For the secondary outcome (Table 4), in the secondary model, blood pH ≤ 7.18 was inversely associated with survival (OR 0.61; 95% CI 0.38–0.98; *p *value = 0.04) while SB use was not. In the interaction analysis, non-SB use × blood pH > 7.18 was positively associated with survival (OR 1.82; 95% CI 1.12–2.95; *p *value = 0.02).

## Discussion

### Main findings

Overall, SB use was not associated with IHCA outcomes when the confounding effects of blood pH were adjusted. Blood pH ≤ 7.18 was significantly associated with worse outcomes. Therefore, by interaction analysis, we tested the hypothesis, i.e. whether the effect of SB would be different in different populations. These populations included patients with blood pH ≤ 7.18 versus > 7.18, patients with versus without severe metabolic acidaemia and patients with CPR duration < versus ≥ 20 min. The results demonstrated that for patients with blood pH > 7.18, non-SB use was associated with survival while for patients with CPR duration ≥ 20 min, SB use was associated with better neurological recovery.

### Comparisons with previous studies

Few studies investigated the effects of SB use during CPR for IHCA^20^. During CPR, point-of-care blood testing may yield important diagnostic information and guide management^21^. Compared with out-of-hospital cardiac arrest (OHCA), it may be easier for clinicians to diagnose acidosis for IHCA. In our study, about 65% (1099 out of 1698) of screened patients had early intra-arrest blood gas analysis, revealing that 10.8% of them were experiencing severe metabolic acidaemia. Nonetheless, not only severe metabolic acidaemia, blood pH ≤ 7.18 itself was identified to be a significant predictor for IHCA outcomes. That is, severe acidosis (blood pH ≤ 7.18)^22,23^, irrespective of the type of acidosis, was more influential on outcomes than pure severe metabolic acidaemia.

Since severe acidosis was both a negative prognostic factor and a potential indication for SB administration^17^, SB use may thus become an epiphenomenon of severe acidosis and could have introduced the bias of “confounding by indication,” leading to worse outcomes associated with SB use^24–27^. When the confounding effects of blood pH were accounted for, the SB use itself was no longer associated with IHCA outcomes, as shown in the Primary Model in the Table 3.

## SB use and intra-arrest blood pH

Furthermore, we attempted to investigate whether the effects of SB would be dependent on intra-arrest blood pH. As shown in Table 3, SB use × blood pH ≤ 7.18 (OR 0.29) and non-SB use × blood pH ≤ 7.18 (OR 0.20) were both associated with worse neurological recovery. Nevertheless, the OR of blood pH ≤ 7.18 itself was 0.24, which was dominant in the interaction terms of SB use × blood pH ≤ 7.18 and non-SB use × blood pH ≤ 7.18 and led to an OR less than one for both interaction terms. Since SB use × blood pH ≤ 7.18 or non-SB use × blood pH ≤ 7.18 was compared with the reference of blood pH > 7.18, it would not be possible for SB use to increase the OR of SB use × blood pH ≤ 7.18 to greater than one. If SB use × blood pH ≤ 7.18 was greater than one, this would mean that the neurological outcomes of patients with blood pH ≤ 7.18 who received SB would be better than those with blood pH > 7.18, which was not expected. Therefore, in the interpretation of this interaction analysis, we should focus on whether SB use could improve the inherently poor neurological outcomes of patients with blood pH ≤ 7.18.

When we examined the two interaction terms, SB use × blood pH ≤ 7.18 (OR 0.29) and non-SB use × blood pH ≤ 7.18 (OR 0.20), we noticed that SB administration actually increased the chances of neurological recovery for patients with blood pH ≤ 7.18 (OR 0.24). The increase in ORs between SB use × blood pH ≤ 7.18 and non-SB use × blood pH ≤ 7.18 was caused by the administration of SB, which suggested that for patients with blood pH ≤ 7.18, SB might improve neurological outcomes. Nonetheless, the effect estimates of SB use × blood pH ≤ 7.18 and non-SB use × blood pH ≤ 7.18 were so close that this result could only indicate a tendency of improvement by SB use rather than a definite conclusion. However, this result may encourage future investigators to examine whether SB use could improve the outcomes of patients with blood pH ≤ 7.18. In contrast, for patients with blood pH > 7.18 (Table 4), non-SB use was associated with better survival.

Taken together, the most important message of this interaction analysis was that the administration of SB had better to be guided by blood pH, instead of being used empirically. The negative effects of SB use noted in previous studies^24–27^ may just reflect that SB administration failed to improve the dismal outcomes of the patients with severe acidosis, rather than worsen it. In contrast, for patients without severe acidosis, SB should not be administered empirically.

### SB use in prolonged CPR

Guidelines for CPR published in the year 2000^28^ suggested that SB could be considered as a last-ditch effort after prolonged CPR had been performed with the confirmed interventions, such as defibrillation, chest compression, intubation, ventilation and vasopressor therapy, already in place and ineffective. As revealed in a paediatric IHCA study^29^, the median CPR duration in the SB group (30 min) was significantly higher than that in the non-SB group (17 min). Andersen et al.^11^ indicated that, if a medication was more likely to be administered when CPR efforts continued longer, this would tend to bias the results toward a harmful effect since CPR duration was strongly associated with worse outcomes^30,31^. This bias might be mitigated to some extent by considering both CPR duration and timing of intervention in the analysis. As shown in Table 3, both CPR duration and time to SB were analysed, revealing that when CPR persisted longer than 20 min, SB use may be beneficial.

The definition of “prolonged CPR” was not clearly defined in the 2000 guidelines for CPR^28^. Vukmir et al.^26^ demonstrated that overall, there was no difference in survival between the SB and placebo groups. Nonetheless, survival rates were significantly higher in the SB group than in the placebo group in the subgroup with prolonged arrest (> 15 min)^23,26^. According to the 3-phase cardiac arrest model^32^, CPR duration longer than 20 min may fall into the metabolic phase, during which interventions other than prompt defibrillation or high-quality CPR should be employed, such as SB. Beyond its metabolic effects, SB was also a volume expander, which might be beneficial during circulatory phase. Nonetheless, since we did not collect the hemodynamic data during CPR, the circulatory effects of SB during CPR should be further examined. Furthermore, despite the fact that our results showed that SB use in prolonged CPR might be effective, it should be remembered that SB is administered after other effective interventions have been performed^28^.

In summary, SB tended to be used in a subgroup with inherently poor outcomes. By carefully dealing with the bias of “confounding by indication” and “resuscitation time bias,” our results seemed to corroborate SB use in certain conditions. However, because of the retrospective study design, the results should be viewed as hypothesis-generating, laying the foundation for further clinical trials.

### Study limitations

First, because of the inherent limitation of the retrospective study design, only an association, rather than a causal relationship, could be investigated between independent and outcome variables. The effects of unmeasured confounders could have introduced bias into the results. Second, it was difficult to ascertain whether blood gas was retrieved from an arterial or a venous origin. It was reported that venous pH may be consistent with arterial pH^33^. However, this could only be resolved by a prospective study adopting a certified method to obtain arterial blood gas. Third, the SB dosage was not accounted for in the analysis. A dose–response effect, if present, could have offered stronger evidence for the effect of SB use on outcomes. Fourth, the exact timing of obtaining blood gas samples was not recorded in the previously established IHCA database^12,13^ and therefore, we could not verify whether the timing of obtaining blood gas samples was prior to the timing of SB use. Nonetheless, since the clinicians in NTUH were instructed to obtain blood gas analysis as soon as possible in the beginning of CPR, the interval between the timing of blood gas analysis and SB use may be quite small (maximum: 10 min), which may not cause significant changes in blood gas analysis data. Finally, we studied only patients with intra-arrest blood gas data available. As shown in Supplemental Table 1 and 2, there were some differences between patients included and not included in the analysis, leading to worse survival and neurological outcomes in patients in current analysis. Therefore, our results may only be applied to patients for whom clinicians would order blood gas analysis. Our results may not be applied to patients for whom clinicians would not order blood gas analysis and also did not suggest that all IHCA patients should receive blood gas analysis. Whether all IHCA patients would benefit from early intra-arrest blood gas analysis should be further examined in prospective studies. Therefore, our results could only be applied to patients who received blood gas analysis during CPR and these results need further examination before they could be generalized to all patients undergoing CPR.

## Conclusions

Our results suggested that SB should not be empirically administered for IHCA patients with blood pH > 7.18. Also, for patients receiving CPR longer than 20 min, SB might be administered, given other effective interventions had been performed.

## Supplementary Information

Supplementary Information 1.

Supplementary Information 2.

Supplementary Information 3.

Supplementary Information 4.

## Data Availability

The data that support the findings of this study are available on request from the corresponding author, Wen-Jone Chen.

## References

[1] Benjamin EJ (2017). Heart disease and stroke statistics-2017 update: A report from the American Heart Association. Circulation.

[2] Lin CC (2018). Association between acidosis and outcome in out-of-hospital cardiac arrest patients. Am. J. Emerg. Med..

[3] Shin J (2017). Initial blood pH during cardiopulmonary resuscitation in out-of-hospital cardiac arrest patients: A multicenter observational registry-based study. Crit. Care.

[4] Wang CH (2020). Associations between early intra-arrest blood acidaemia and outcomes of adult in-hospital cardiac arrest: A retrospective cohort study. J. Formos. Med. Assoc..

[5] Standards for Cardiopulmonary Resuscitation (CPR) and Emergency Cardiac Care (ECC). *JAMA* **227**, 833–68 (1974). 10.1001/jama.227.7.833 28834958

[6] Soar J (2015). European resuscitation council guidelines for resuscitation 2015: Section 3. Adult advanced life support. Resuscitation.

[7] Link MS (2015). Part 7: Adult Advanced Cardiovascular Life Support: 2015 American Heart Association Guidelines Update for Cardiopulmonary Resuscitation and Emergency Cardiovascular Care. Circulation.

[8] Moskowitz A (2019). Trends over time in drug administration during adult in-hospital cardiac arrest. Crit. Care Med..

[9] Geraci MJ (2009). Prevalence of sodium bicarbonate-induced alkalemia in cardiopulmonary arrest patients. Ann. Pharmacother.

[10] Bar-Joseph G (2002). Clinical use of sodium bicarbonate during cardiopulmonary resuscitation–is it used sensibly?. Resuscitation.

[11] Andersen LW, Grossestreuer AV, Donnino MW (2018). "Resuscitation time bias"-A unique challenge for observational cardiac arrest research. Resuscitation.

[12] Wang CH (2018). Associations between body size and outcomes of adult in-hospital cardiac arrest: A retrospective cohort study. Resuscitation.

[13] Wang CH (2018). The association between long-term glycaemic control, glycaemic gap and neurological outcome of in-hospital cardiac arrest in diabetics: A retrospective cohort study. Resuscitation.

[14] ECC Committee, Subcommittees and Task Forces of the American Heart Association. 2005 American Heart Association Guidelines for Cardiopulmonary Resuscitation and Emergency Cardiovascular Care. *Circulation* **112**, IV1–203 (2005). 10.1161/CIRCULATIONAHA.105.166550 16314375

[15] Field JM (2010). Part 1: Executive summary: 2010 American Heart Association Guidelines for Cardiopulmonary Resuscitation and Emergency Cardiovascular Care. Circulation.

[16] Jacobs I (2004). Cardiac arrest and cardiopulmonary resuscitation outcome reports: Update and simplification of the Utstein templates for resuscitation registries: A statement for healthcare professionals from a task force of the International Liaison Committee on Resuscitation (American Heart Association, European Resuscitation Council, Australian Resuscitation Council, New Zealand Resuscitation Council, Heart and Stroke Foundation of Canada, InterAmerican Heart Foundation, Resuscitation Councils of Southern Africa). Circulation.

[17] Jaber S (2018). Sodium bicarbonate therapy for patients with severe metabolic acidaemia in the intensive care unit (BICAR-ICU): A multicentre, open-label, randomised controlled, phase 3 trial. Lancet.

[18] Becker LB (2011). Primary outcomes for resuscitation science studies: A consensus statement from the American Heart Association. Circulation.

[19] Hastie TJ, Tibshirani RJ (1990). Generalized Additive Models.

[20] Velissaris D (2016). Use of sodium bicarbonate in cardiac arrest: Current guidelines and literature review. J. Clin. Med. Res..

[21] Wang CH (2016). The effects of calcium and sodium bicarbonate on severe hyperkalaemia during cardiopulmonary resuscitation: A retrospective cohort study of adult in-hospital cardiac arrest. Resuscitation.

[22] Kraut JA, Kurtz I (2006). Use of base in the treatment of acute severe organic acidosis by nephrologists and critical care physicians: Results of an online survey. Clin. Exp. Nephrol..

[23] Kraut JA, Madias NE (2010). Metabolic acidosis: Pathophysiology, diagnosis and management. Nat. Rev. Nephrol..

[24] van Walraven C (1998). Do advanced cardiac life support drugs increase resuscitation rates from in-hospital cardiac arrest? The OTAC Study Group. Am. J. Emerg. Med..

[25] Aufderheide TP (1992). Prehospital bicarbonate use in cardiac arrest: A 3-year experience. Am. J. Emerg. Med..

[26] Vukmir RB, Katz L, Sodium Bicarbonate Study Group (2006). Sodium bicarbonate improves outcome in prolonged prehospital cardiac arrest. Am. J. Emerg. Med..

[27] Kawano T (2017). Prehospital sodium bicarbonate use could worsen long term survival with favorable neurological recovery among patients with out-of-hospital cardiac arrest. Resuscitation.

[28] Part 6: Advanced Cardiovascular Life Support. *Circulation* **102**, I-129-I-135 (2000).

[29] Raymond TT (2015). Sodium bicarbonate use during in-hospital pediatric pulseless cardiac arrest—A report from the American Heart Association Get With The Guidelines^®^-Resuscitation. Resuscitation.

[30] Chan PS (2012). A validated prediction tool for initial survivors of in-hospital cardiac arrest. Arch. Intern. Med..

[31] Wang CH (2018). Validation of the Cardiac Arrest Survival Postresuscitation In-hospital (CASPRI) score in an East Asian population. PLoS One.

[32] Weisfeldt ML, Becker LB (2002). Resuscitation after cardiac arrest: A 3-phase time-sensitive model. JAMA.

[33] Rang LC (2002). Can peripheral venous blood gases replace arterial blood gases in emergency department patients?. CJEM.

