# Supplementary material for: A retrospective study on the therapeutic effects of sodium bicarbonate for adult in-hospital cardiac arrest
Source: Sci Rep. 2021 Jun 11;11:12380. doi: 10.1038/s41598-021-91936-3 (PMC8196083; doi:10.1038/s41598-021-91936-3)
Supplement: Supplementary file 1 — Supplementary Information 1. [file 41598_2021_91936_MOESM1_ESM.docx]

**A Retrospective Study on the Blood pH- and Timing-dependent Effects of Sodium Bicarbonate for Adult In-Hospital Cardiac Arrest**

Chih-Hung Wang, MD, PhD^1,2^; Cheng-Yi Wu, MD^1^; Meng-Che Wu, MD^1^; Wei-Tien Chang, MD, PhD^1,2^; Chien-Hua Huang, MD, PhD^1,2^; Min-Shan Tsai, MD, PhD^1,2^; Tsung-Chien Lu, MD, PhD^1,2^; Eric Chou, MD^3^; Yu-Lin Hsieh, MD^4^; Wen-Jone Chen, MD, PhD^1,2,5,*^

Supplemental Table 1. Baseline Characteristics of Patients Stratified by Inclusion Status

| Variables | Patients included in the analysis (n=1060) | Patients excluded from the analysis (n=638) | *p*-value |
| --- | --- | --- | --- |
| Age, years (SD) | 68.2 (56.5-78.9) | 65.1 (51.9-77.0) | 0.003 |
| Male, n (%) | 649 (61.2) | 387 (60.7) | 0.82 |
| Comorbidities, n (%) |  |  |  |
| Heart failure, this admission | 208 (19.6) | 120 (18.8) | 0.68 |
| Heart failure, prior admission | 171 (16.1) | 103 (16.1) | 0.99 |
| Myocardial infarction, this admission | 120 (11.3) | 96 (15) | 0.03 |
| Myocardial infarction, prior admission | 39 (3.7) | 34 (5.3) | 0.10 |
| Arrhythmia | 192 (18.1) | 114 (17.9) | 0.90 |
| Hypotension | 260 (24.5) | 157 (24.6) | 0.97 |
| Respiratory insufficiency | 764 (72.1) | 460 (72.1) | 0.99 |
| Renal insufficiency | 446 (42.1) | 252 (39.5) | 0.30 |
| Hepatic insufficiency | 182 (17.2) | 123 (19.3) | 0.27 |
| Metabolic or electrolyte  abnormality | 186 (17.5) | 98 (15.4) | 0.24 |
| Diabetes mellitus | 354 (33.4) | 202 (31.7) | 0.46 |
| Baseline evidence of motor, cognitive, or functional deficits | 329 (31.0) | 209 (32.8) | 0.46 |
| Acute stroke | 45 (4.2) | 30 (4.7) | 0.66 |
| Favourable neurological status 24 h before cardiac arrest | 462 (43.6) | 275 (43.1) | 0.85 |
| Pneumonia | 337 (31.8) | 203 (31.8) | 0.99 |
| Bacteraemia | 86 (8.1) | 58 (9.1) | 0.48 |
| Cirrhosis | 70 (6.6) | 46 (7.2) | 0.63 |
| Chronic obstructive pulmonary disease | 62 (5.8) | 27 (4.2) | 0.15 |
| Dialysis | 191 (18) | 103 (16.1) | 0.32 |
| Metastatic cancer or any blood-borne malignancy | 247 (23.3) | 141 (22.1) | 0.57 |
| Charlson comorbidity index (SD) | 2 (1-4) | 2 (1-4) | 0.38 |

Abbreviations: SD, standard deviation
