# Supplementary material for: A retrospective study on the therapeutic effects of sodium bicarbonate for adult in-hospital cardiac arrest
Source: Sci Rep. 2021 Jun 11;11:12380. doi: 10.1038/s41598-021-91936-3 (PMC8196083; doi:10.1038/s41598-021-91936-3)
Supplement: Supplementary file 2 — Supplementary Information 2. [file 41598_2021_91936_MOESM2_ESM.docx]

**A Retrospective Study on the Blood pH- and Timing-dependent Effects of Sodium Bicarbonate for Adult In-Hospital Cardiac Arrest**

Chih-Hung Wang, MD, PhD^1,2^; Cheng-Yi Wu, MD^1^; Meng-Che Wu, MD^1^; Wei-Tien Chang, MD, PhD^1,2^; Chien-Hua Huang, MD, PhD^1,2^; Min-Shan Tsai, MD, PhD^1,2^; Tsung-Chien Lu, MD, PhD^1,2^; Eric Chou, MD^3^; Yu-Lin Hsieh, MD^4^; Wen-Jone Chen, MD, PhD^1,2,5,*^

Supplemental Table 2. Features, Interventions and Outcomes of Cardiac Arrest Events Stratified by Inclusion Status

| Variables | Patients included in the analysis (n=1060) | Patients excluded from the analysis (n=638) | | *p*-value |
| --- | --- | --- | --- | --- |
| Arrest at night, n (%) | 385 (36.3) | 179 (27.1) | | <0.001 |
| Arrest on weekend, n (%) | 301 (28.4) | 184 (28.8) | | 0.84 |
| Arrest location, n (%) |  |  | | 0.14 |
| Intensive care unit | 473 (44.6) | 306 (48) | |  |
| General ward | 525 (49.5) | 286 (44.8) | |  |
| Others | 62 (5.8) | 46 (7.2) | |  |
| Witnessed arrest, n (%) | 734 (69.2) | 468 (73.4) | | 0.07 |
| Monitored status, n (%) | 645 (60.8) | 412 (64.6) | | 0.13 |
| Shockable rhythm, n (%) | 147 (13.9) | 119 (18.7) | | 0.009 |
| Critical care interventions in place at time of arrest, n (%) |  |  | |  |
| Mechanical ventilation | 267 (25.2) | 143 (22.4) | | 0.20 |
| Antiarrhythmics | 119 (11.2) | 78 (12.2) | | 0.53 |
| Vasopressors | 474 (44.7) | 286 (44.8) | | 0.96 |
| Dialysis | 78 (7.4) | 45 (7.1) | | 0.81 |
| Pulmonary artery catheter | 6 (0.6) | 8 (1.3) | | 0.13 |
| Intra-aortic balloon pumping | 8 (0.8) | 11 (1.7) | | 0.07 |
| CPR duration, min (SD) | 30 (14-49) | 20 (8-37) | | <0.001 |
| SB use, n (%) | 733 (69.2) | 308 (48.3) | | <0.001 |
| Post-ROSC interventions, n (%) |  |  | |  |
| Extracorporeal membrane oxygenation | 84 (7.9) | 55 (8.6) | | 0.61 |
| Targeted temperature management | 12 (1.1) | 1 (0.2) | | 0.03 |
| Percutaneous coronary intervention | 35 (3.3) | | 45 (7.1) | <0.001 |
| Sustained ROSC, n (%) | 584 (55.1) | | 386 (60.5) | 0.03 |
| Survival to hospital discharge, n (%) | 124 (11.7) | | 116 (18.2) | <0.001 |
| Favourable Neurological Outcome at Hospital Discharge, n (%) | 59 (5.6) | | 65 (10.2) | <0.001 |

Abbreviations: CPR, cardiopulmonary resuscitation; SD, standard deviation; SB, sodium bicarbonate; ROSC, return of spontaneous circulation
