# Supplementary material for: A retrospective study on the therapeutic effects of sodium bicarbonate for adult in-hospital cardiac arrest
Source: Sci Rep. 2021 Jun 11;11:12380. doi: 10.1038/s41598-021-91936-3 (PMC8196083; doi:10.1038/s41598-021-91936-3)
Supplement: Supplementary file 3 — Supplementary Information 3. [file 41598_2021_91936_MOESM3_ESM.docx]

**A Retrospective Study on the Blood pH- and Timing-dependent Effects of Sodium Bicarbonate for Adult In-Hospital Cardiac Arrest**

Chih-Hung Wang, MD, PhD^1,2^; Cheng-Yi Wu, MD^1^; Meng-Che Wu, MD^1^; Wei-Tien Chang, MD, PhD^1,2^; Chien-Hua Huang, MD, PhD^1,2^; Min-Shan Tsai, MD, PhD^1,2^; Tsung-Chien Lu, MD, PhD^1,2^; Eric Chou, MD^3^; Yu-Lin Hsieh, MD^4^; Wen-Jone Chen, MD, PhD^1,2,5,*^

Supplemental Table 3. Baseline Characteristics of Patients Stratified by Administration of Sodium Bicarbonate

| Variables | Patients with administration of SB (n=733) | Patients without administration of SB (n=327) | *p*-value |
| --- | --- | --- | --- |
| Age, years (SD) | 68.2 (57.2-79.3) | 67.9 (55.4-78.5) | 0.50 |
| Male, n (%) | 444 (60.6) | 205 (62.7) | 0.51 |
| Comorbidities, n (%) |  |  |  |
| Heart failure, this admission | 145 (19.8) | 63 (19.3) | 0.85 |
| Heart failure, prior admission | 120 (16.4) | 51 (15.6) | 0.75 |
| Myocardial infarction, this admission | 82 (11.2) | 38 (11.6) | 0.84 |
| Myocardial infarction, prior admission | 24 (3.3) | 15 (4.6) | 0.29 |
| Arrhythmia | 132 (18) | 60 (18.3) | 0.89 |
| Hypotension | 175 (23.9) | 85 (26.0) | 0.46 |
| Respiratory insufficiency | 525 (71.6) | 239 (73.1) | 0.62 |
| Renal insufficiency | 313 (42.7) | 133 (40.7) | 0.54 |
| Hepatic insufficiency | 123 (16.8) | 59 (18.0) | 0.61 |
| Metabolic or electrolyte  abnormality | 121 (16.5) | 65 (19.9) | 0.18 |
| Diabetes mellitus | 244 (33.3) | 110 (33.6) | 0.91 |
| Baseline evidence of motor, cognitive, or functional deficits | 240 (32.7) | 89 (32.7) | 0.07 |
| Acute stroke | 26 (3.5) | 19 (5.8) | 0.09 |
| Favourable neurological status 24 h before cardiac arrest | 325 (44.3) | 137 (41.9) | 0.46 |
| Pneumonia | 227 (31) | 110 (33.6) | 0.39 |
| Bacteraemia | 57 (7.8) | 29 (8.9) | 0.55 |
| Cirrhosis | 43 (5.9) | 27 (8.3) | 0.15 |
| Chronic obstructive pulmonary disease | 43 (5.9) | 19 (5.8) | 0.97 |
| Dialysis | 137 (18.7) | 54 (16.5) | 0.39 |
| Metastatic cancer or any blood-borne malignancy | 164 (22.4) | 83 (25.4) | 0.28 |
| Charlson comorbidity index (SD) | 2 (1-4) | 2 (1-5) | 0.38 |

Abbreviations: SD, standard deviation
