# Supplementary material for: A retrospective study on the therapeutic effects of sodium bicarbonate for adult in-hospital cardiac arrest
Source: Sci Rep. 2021 Jun 11;11:12380. doi: 10.1038/s41598-021-91936-3 (PMC8196083; doi:10.1038/s41598-021-91936-3)
Supplement: Supplementary file 4 — Supplementary Information 4. [file 41598_2021_91936_MOESM4_ESM.docx]

**A Retrospective Study on the Blood pH- and Timing-dependent Effects of Sodium Bicarbonate for Adult In-Hospital Cardiac Arrest**

Chih-Hung Wang, MD, PhD^1,2^; Cheng-Yi Wu, MD^1^; Meng-Che Wu, MD^1^; Wei-Tien Chang, MD, PhD^1,2^; Chien-Hua Huang, MD, PhD^1,2^; Min-Shan Tsai, MD, PhD^1,2^; Tsung-Chien Lu, MD, PhD^1,2^; Eric Chou, MD^3^; Yu-Lin Hsieh, MD^4^; Wen-Jone Chen, MD, PhD^1,2,5,*^

Supplemental Table 4. Features, Interventions and Outcomes of Cardiac Arrest Events Stratified by Administration of Sodium Bicarbonate

| Variables | Patients with administration of SB (n=733) | Patients without administration of SB (n=327) | | *p*-value |
| --- | --- | --- | --- | --- |
| Arrest at night, n (%) | 289 (39.4) | 96 (29.4) | | 0.001 |
| Arrest on weekend, n (%) | 210 (28.6) | 97 (27.8) | | 0.78 |
| Arrest location, n (%) |  |  | | 0.02 |
| Intensive care unit | 310 (42.3) | 163 (49.8) | |  |
| General ward | 384 (52.4) | 141 (43.1) | |  |
| Others | 39 (5.3) | 23 (7.0) | |  |
| Witnessed arrest, n (%) | 494 (67.4) | 240 (73.4) | | 0.05 |
| Monitored status, n (%) | 433 (59.1) | 212 (64.8) | | 0.08 |
| Shockable rhythm, n (%) | 89 (12.1) | 58 (17.7) | | 0.02 |
| Critical care interventions in place at time of arrest, n (%) |  |  | |  |
| Mechanical ventilation | 194 (26.5) | 73 (22.3) | | 0.15 |
| Antiarrhythmics | 88 (12) | 31 (9.5) | | 0.23 |
| Vasopressors | 325 (44.3) | 149 (45.6) | | 0.71 |
| Dialysis | 53 (7.2) | 25 (7.6) | | 0.81 |
| Pulmonary artery catheter | 3 (0.4) | 3 (0.9) | | 0.31 |
| Intra-aortic balloon pumping | 7 (1) | 1 (0.3) | | 0.26 |
| CPR duration, min (SD) | 35 (20-56) | 14 (7.3-31) | | <0.001 |
| Post-ROSC interventions, n (%) |  |  | |  |
| Extracorporeal membrane oxygenation | 60 (8.2) | 24 (7.3) | | 0.64 |
| Targeted temperature management | 10 (1.4) | 2 (0.6) | | 0.28 |
| Percutaneous coronary intervention | 21 (2.9) | | 14 (4.3) | 0.23 |
| Sustained ROSC, n (%) | 355 (48.4) | | 229 (70) | <0.001 |
| Survival to hospital discharge, n (%) | 52 (7.1) | | 72 (22) | <0.001 |
| Favourable Neurological Outcome at Hospital Discharge, n (%) | 27 (3.7) | | 32 (9.8) | <0.001 |

Abbreviations: CPR, cardiopulmonary resuscitation; SD, standard deviation; SB, sodium bicarbonate; ROSC, return of spontaneous circulation
